# Supplementary material for: Acidity Suppression of Hole Transport Layer via Solution Reaction of Neutral PEDOT:PSS for Stable Perovskite Photovoltaics
Source: Polymers (Basel). 2020 Jan 6;12(1):129. doi: 10.3390/polym12010129 (PMC7022435; doi:10.3390/polym12010129)
Supplement: Supplementary file 1 [file polymers-12-00129-s001.pdf]

Polymers **2020**, *12*, 129; doi:10.3390/polym12010129

(Supplementary Information)

Acidity Suppression of Hole Transport Layer via Solution Reaction of Neutral PEDOT:PSS for Stable Perovskite Photovoltaics

Minseong Kim[1], Minji Yi[1], Woongsik Jang[1], Jung Kyu Kim[2,*] and Dong Hwan Wang[1,*]

[1] School of Integrative Engineering, Chung-Ang University, Seoul 06974, Republic of Korea;
[2] School of Chemical Engineering, Sungkyunkwan University (SKKU), Suwon-si 16419, Republic of Korea;
* Correspondence: legkim@skku.edu (Prof. J.K. Kim); Tel.: +82-31-290-7254, and king0401@cau.ac.kr (Prof. D. H. Wang); Tel.: +82-2-820-5074

Keywords: Conducting Polymers, Photovoltaic Devices, Controlled pH, Charge Transport, Stability

Table S1. Peak integration and ratio of various PEDOT:PSS typical bands in the FTIR spectrum

	Imidazole (C- N) 1277 cm^{-1}	PSS (O-S-O) 1003 cm^{-1}	PEDOT (C-S-C) 685 cm^{-1}
AN10	0	8.78	13.78
AN11	8.27	17.3	28.64
AN13	14.35	25.58	30.05

Table S2. Electrical parameters of the neutral PEDOT:PSS-based PSCs

Acid:Neutral	Voc (V)	Jsc (mA/cm2)	IPCE (mA/cm2)	FF (%)	PCE (%)
AN01 (Neutral)	0.876	14.36	13.85	54	6.74

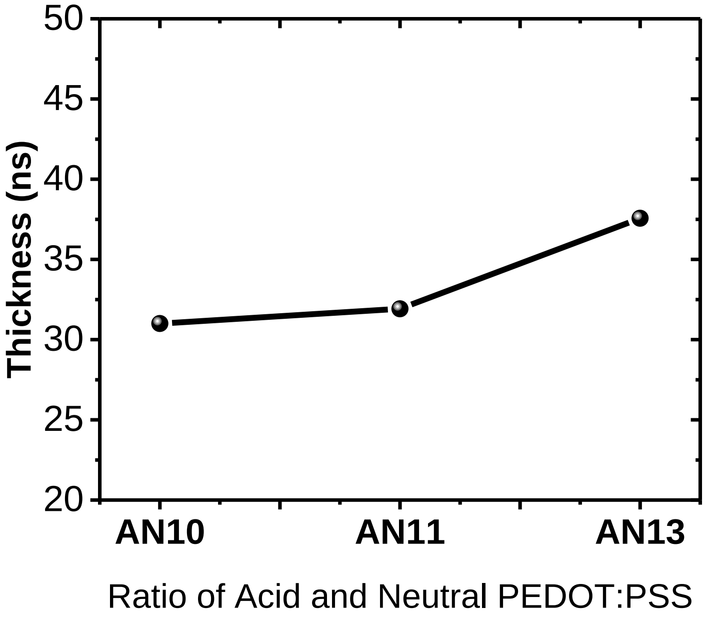

Figure S1. Thickness of spin-coated AN10-, AN11-, and AN13-PEDOT:PSS films obtained by the Dektak XT thickness profiler

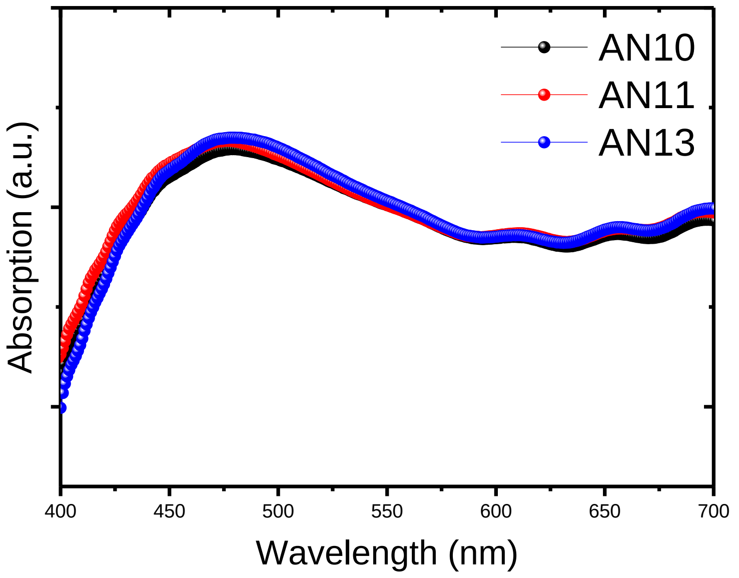

Figure S2. Absorbance of the spin-coated AN10-, AN11-, and AN13-PEDOT:PSS films

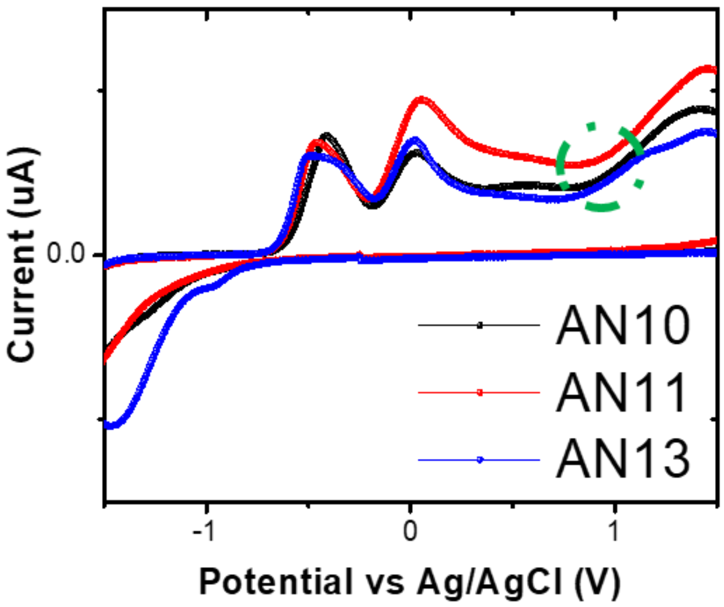

Figure S3. Cyclic voltammetry (first scans) curves of AN10-, AN11-, and AN13-PEDOT:PSS films deposited onto a Pt disk electrode in Bu_4NPF_6/acetonitrile supporting an electrolyte/solvent system

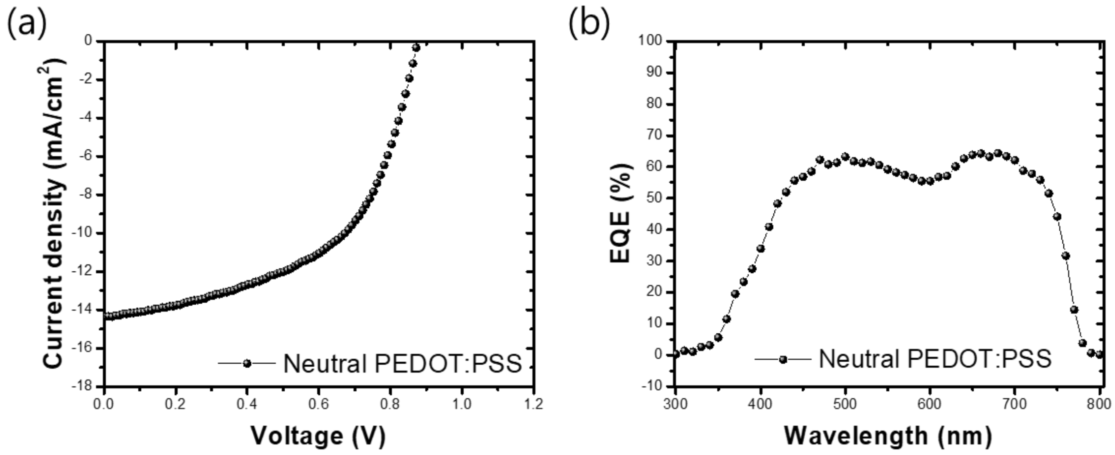

Figure S4. (a) Current-voltage (J-V) characteristics of neutral PEDOT:PSS-based PSCs under AM 1.5 irradiation at 100 mW cm^{-2}. (b) EQE of neutral PEDOT:PSS-based PSCs depending on J-V characteristics.
